# Enhancing Autophagy Diminishes Aberrant Ca^2+^ Homeostasis and Arrhythmogenesis in Aging Rabbit Hearts

**DOI:** 10.3389/fphys.2019.01277

**Published:** 2019-10-04

**Authors:** Kevin R. Murphy, Brett Baggett, Leroy L. Cooper, Yichun Lu, Jin O-Uchi, John M. Sedivy, Dmitry Terentyev, Gideon Koren

**Affiliations:** ^1^Cardiovascular Research Center at the Cardiovascular Institute, Division of Cardiology, Warren Alpert Medical School of Brown University, Providence, RI, United States; ^2^Department of Molecular Biology, Cellular Biology and Biochemistry, Brown University, Providence, RI, United States; ^3^Department of Biology, Vassar College, Poughkeepsie, NY, United States

**Keywords:** autophagy, aging, calcium, cardiac physiology, ryanodine receptor

## Abstract

**Aim:**

Aging in humans is associated with a 10–40-fold greater incidence of sudden cardiac death from malignant tachyarrhythmia. We have reported that thiol oxidation of ryanodine receptors (RyR2s) by mitochondria-derived reactive oxygen species (mito-ROS) contributes to defective Ca^2+^ homeostasis in cardiomyocytes (CMs) from aging rabbit hearts. However, mechanisms responsible for the increase in mito-ROS in the aging heart remain poorly understood. Here we test the hypothesis that age-associated decrease in autophagy is a major contributor to enhanced mito-ROS production and thereby pro-arrhythmic disturbances in Ca^2+^ homeostasis.

**Methods and Results:**

Ventricular tissues from aged rabbits displayed significant downregulation of proteins involved in mitochondrial autophagy compared with tissues from young controls. Blocking autophagy with chloroquine increased total ROS production in primary rabbit CMs and mito-ROS production in HL-1 CMs. Furthermore, chloroquine treatment of HL-1 cells depolarized mitochondrial membrane potential (Δψm) to 50% that of controls. Blocking autophagy significantly increased oxidation of RyR2, resulting in enhanced propensity to pro-arrhythmic spontaneous Ca^2+^ release under β-adrenergic stimulation. Aberrant Ca^2+^ release was abolished by treatment with the mito-ROS scavenger mito-TEMPO. Importantly, the autophagy enhancer Torin1 and ATG7 overexpression reduced the rate of mito-ROS production and restored both Δψm and defective Ca^2+^ handling in CMs derived from aged rabbit hearts.

**Conclusion:**

Decreased autophagy is a major cause of increased mito-ROS production in the aging heart. Our data suggest that promoting autophagy may reduce pathologic mito-ROS during normal aging and reduce pro-arrhythmic spontaneous Ca^2+^ release via oxidized RyR2s.

## Introduction

Sudden cardiac death due to malignant ventricular tachyarrhythmia remains a major cause of mortality in the United States, with ∼300,000 cases each year (Deo and Albert, 2012). Risk of sudden cardiac death increases 5–40-fold in people over 65 years old compared to younger individuals (Zheng et al., 2001; Podrid and Myerburg, 2005). We used the aged rabbit model to study the mechanisms of arrhythmogenesis in aging humans. The rabbit heart closely resembles the human heart in its electrical and contractile properties (Cooper et al., 2012). Our studies of aged rabbit hearts (>4 years old) revealed conduction abnormalities, myocardial stiffening, increased interstitial fibrosis, slowed transverse conduction, and depletion of the Purkinje network (Cooper et al., 2012). Moreover, we demonstrated that aged rabbit cardiomyocytes (CMs) have depolarized mitochondrial membrane potentials (Δψm), higher levels of mitochondria-derived reactive oxygen species (mito-ROS), increased calcium (Ca^2+^) leak from the mito-ROS-oxidized type 2 ryanodine receptor (RyR2), and increased formation of pro-arrhythmic spontaneous Ca^2+^ waves (SCWs) (Cooper et al., 2013). Mitochondria participate in a variety of intracellular tasks including energy production and nutrient sensing (Dai et al., 2012). Excessive production of ROS by defective mitochondria is proarrhythmogenic and predisposes the aging heart to lethal ventricular tachyarrhythmia (Zorov et al., 2000; Janczewski and Lakatta, 2010; Cooper et al., 2013; Hamilton and Terentyev, 2019).

Intracellular Ca^2+^ release from the SR mediated by RyR2 is a critical determinant of cardiac contractility. Abnormally high RyR2 activity has been implicated in arrhythmogenesis and is present in a variety of diseases such as heart failure and myocardial infarction (Wehrens et al., 2005; Belevych et al., 2011, 2012; Plummer et al., 2011; Zima et al., 2014). We have demonstrated that hyperactivity of RyR2 in aged hearts is caused by oxidation of reactive cysteines in the channel by excessive ROS emitted by mitochondria and that treatment of aged myocytes with the mitochondria-specific scavenger mito-TEMPO effectively restores intracellular Ca^2+^ cycling (Cooper et al., 2013). However, the mechanisms underlying enhanced mito-ROS generation and concomitant pro-arrhythmic changes in Ca^2+^ cycling, characteristic of aging, are not well understood.

The mitochondrial population is a vast network controlled by cycles of fission and fusion and is regulated by mitophagy or autophagy at the organelle level. Fission is a culling mechanism for damaged mitochondria, while fusion recombines fragmented, healthy mitochondria into larger, bio-energetically favorable structures. Autophagy sequesters unnecessary or dysfunctional cellular components (including proteins, organelles, and even bacteria) in a double-membrane structure called an autophagosome that is then degraded by lysosomal acidic hydrolases (Ravikumar et al., 2009; Ashrafi and Schwarz, 2013). Damaged smaller mitochondria produced by fission are cleared from the mitochondrial network by mitophagy, which is a self-degradative catabolic process (Kubli and Gustafsson, 2012; Ashrafi and Schwarz, 2013). Mitophagy involves upregulation of general autophagy machinery components followed by the recognition of damaged mitochondria as targets for clearance. Without efficient autophagy (and its mitochondria-specific form, mitophagy), damaged mitochondria would remain in the cell and generate harmful ROS (Miquel et al., 1980; Terman et al., 2010; Sharp et al., 2014). Because both autophagy and mitophagy are impaired in the aged heart (Taneike et al., 2010; Dai et al., 2012; Lopez-Otin et al., 2013), we hypothesized that decreased autophagy may predispose the aging heart to arrhythmogenesis.

Here we show that inhibition of autophagy with chloroquine, which blocks lysosomal acidification and prevents autophagosome fusion and therefore subsequent degradation (Shintani and Klionsky, 2004), promoted generation of SCWs at the cellular scale because of RyR2 oxidation. By contrast, enhancing autophagy in cultured primary aged myocytes using Torin1, a potent inhibitor of the negative autophagy regulator mTOR (Thoreen et al., 2009), reduced the frequency of spontaneous Ca^2+^ release. In addition to pharmacologic experiments, to enhance autophagy we overexpressed ATG7, an essential component of the autophagosome that is sufficient to increase autophagy flux (Bhuiyan et al., 2013; Martinez-Lopez et al., 2015). The results of these overexpression experiments showed a significant reduction in age-related instances of spontaneous Ca^2+^ release. We conclude that defective autophagy contributes to the age-associated increase in mito-ROS production and pro-arrhythmic Ca^2+^ mishandling.

## Materials and Methods

### Primary Cell Culture and Infection

Primary rabbit CMs were isolated from young (4.5–9 months) or aged (>4 years) New Zealand White rabbits (RSI Farms, Mocksville, NC, United States) as previously described, by Langendorff perfusion with a solution containing collagenase II (#CLS-2, Worthington Biochemical, Lakewood, NJ, United States) (Cooper et al., 2013). Myocytes were plated for 1 h in M199 (#M4530, Sigma, St. Louis, MO, United States) supplemented with 10% fetal bovine serum, penicillin (100 μ/mL), and streptomycin (0.1 μ/mL) (#P4333, Sigma, St. Louis, MO, United States). HL-1 CMs were cultured as previously described (Claycomb et al., 1998). Adenovirus infections of cells were performed at MOI = 1 unless otherwise indicated. Ca^2+^ handling experiments were performed ≥36 h post-infection. All other assays were performed 48 h post-infection unless otherwise indicated. Ratiometric assessment of mito-H_2_O_2_ was performed 5 days after infection in HL-1 CMs using the genetically encoded MTroGFP-ORP1 sensor as previously described (Dey et al., 2018).

### Transmission Electron Microscopy of Primary Isolated Ventricular Rabbit Myocytes

Primary rabbit left ventricular CMs were plated on Permanox Lab-Tek chamber slides and fixed with 1.25% glutaraldehyde in 0.15 M sodium cacodylate buffer. Cells were post-fixed with 1% osmium tetroxide, stained with 2% uranyl acetate, and dehydrated via graded ethanol series. Slides were covered with Epox 812 resin and polymerized overnight. Selected areas were cut and mounted for sectioning. Ultra-thin sections (50–60 nm) were prepared using a Reichert Ultracut S microtome, retrieved onto 300 mesh copper grids, and contrasted with uranyl acetate and lead citrate. Images were collected on a Morgagni 268 transmission electron microscope with AMT Advantage 542 CCD camera system. Mitochondrial area and aspect ratio were analyzed using the publicly available ImageJ.

### Western Blotting of Autophagy and Mitochondrial Proteins

Protein expression was determined using the following antibodies in immunoblotting: p62 1:1000 (Abcam #ab56416), PINK1 1:1000 (Cell Signaling #9646), p53 (Abcam #ab17869), LC3 (Abgent # AM1800A), DRP1 1:500 (Abcam #AB56788), and MFN2 1:500 (Sigma #WH0009927M3). Left ventricular free-wall tissue was homogenized in protein lysis buffer (50 mM Tris–HCL pH 7.5, 150 mM NaCl, 1 mM Ethylenediaminetetraacetic acid (EDTA), 10% glycerol, 1 mM Phenylmethylsulfonyl fluoride, 1 tablet Roche Complete MINI protease inhibitor). Samples were centrifuged at 2.5 k RPM for 15 min, aliquoted, and stored at −80°C. A total of 15–25 μg protein was loaded into 4–15% TGX gels (#4561086, BioRad, Hercules, CA, United States) via SDS-PAGE, transferred onto Polyvinylidene difluoride (PVDF) membranes, and probed with mouse or rabbit antibodies specific for these proteins and subsequently probed with donkey anti-mouse or anti-rabbit secondary antibodies (Thermo Fisher, Waltham, MA, United States). Blots were developed with SuperSignal West Pico (#34080, Thermo Fisher, Waltham, MA, United States), imaged with ChemiDoc 2000 (BioRad, Hercules, CA, United States), and quantified and analyzed using ImageLab (BioRad, Hercules, CA, United States) and ImageJ (US National Institutes of Health, Bethesda, MD, United States) softwares.

### SDS–PAGE and Western Blotting for RyR2 Oxidation Using C2-Maleimide

Radioimmunoprecipitation assay buffer extraction buffer (50 mM Tris–HCL pH 7.5, 100 mM NaCl, 10 mM EDTA, 1% Triton X-100, 1% NP-40, 0.2% SDS, 1 tablet Roche Complete MINI protease inhibitor, pH 7.5) was added to cell culture plates, scraped, collected, and kept on ice for 45 min with vortexing every 15 min. Lysates were spun at 4°C for 30 min at 5,800 × *g*. 25 μL of concentrated supernatant was treated with 40 μL of 0.225 mM Alexa 647 C2-maleimide and incubated for 2 h at room temperature in the dark, with rotation. 450 μL of ice-cold acetone was added and incubated for 1 h at −20°C. The protein pellet was resuspended in 1× Laemmli buffer (#161-0737, BioRad, Hercules, CA, United States) and loaded into a 4–15% TGX gel (#4561086, BioRad, Hercules, CA, United States). Gels were imaged with ChemiDoc-2000 (BioRad, Hercules, CA, United States) using the Alexa 647 preset. Protein was then transferred onto PVDF membranes and probed with anti-RyR 1:1000 (#MA3-916, Thermo Fisher, Waltham, MA, United States) and donkey anti-mouse secondary antibody (#PA1-28748, Thermo Fisher, Waltham, MA, United States). Blots were developed as described above.

### Fluorescence Microscopy Measurements of Autophagy Flux Using a TF-LC3-GFP Biosensor

Autophagy flux was measured using the tandem fluorescence LC3-GFP adenovirus. CMs were measured in Tyrode solution (140 mM NaCl, 5.4 mM KCl, 1.8 mM Ca^2+^,1 mM MgCl_2_, 10 mM HEPES, 5.6 mM glucose, pH = 7.3 using NaOH). Cells were infected for 48 h with 1 MOI of adenovirus containing TF-LC3-GFP. Z-stack images were taken in 1-μm steps through the cell. Two channels per stack were collected. GFP was excited using a 460–500 nm filter and emission was collected from 500 to 560 nm. RFP was excited using a 540–580 nm filter and emission was collected at 600–660 nm. Focused images were created in NIS Elements V3 (Nikon Instruments). Pixels ratio = 1 represent the autolysosomal region. Total areas of ratio >1 were calculated with respect to total surface area to measure autophagy flux.

### Confocal Microscopy Measurements of Intracellular ROS and Δψm

Measurements of intracellular ROS and mitochondrial membrane potential (Δψm) were measured on a Leica SP5 confocal microscope using a 60 × 1.4 NA oil immersion objective in XYT mode. ROS production by CMs was measured in Tyrode solution (see above) using the ROS-sensitive dye 5-(and-6)-chloromethyl-2′,7′-dichlorodihydrofluorescein diacetate (CM-H2DCFDA, 7.5 μM, incubated for 20 min) as previously described (Cooper et al., 2013). DCFDA dye was excited at 488 nm with an argon laser in XY mode, and emission was collected at 500–530 nm. The rate of ROS production was measured in CMs *in vitro* and *ex vivo* as well as those pretreated with mito-TEMPO (25 μM for 10 min). Mitochondrial ROS production was measured in Tyrode solution using the mitochondria superoxide-sensitive fluorescent indicator MitoSOX Red (1 μM, incubated in cell culture medium for 30 min). The dye was excited at 514 nm with a HeNe laser in XY mode, and emission was collected at 640–660 nm. Mitochondrial membrane potential was monitored with the voltage-sensitive fluorescent indicator tetramethylrhodamine methyl ester (TMRM) as previously described (Cordeiro et al., 2015). Briefly, CMs were loaded with 1 nM TMRM (10 min), and TMRM fluorescence was measured in XY mode. TMRM was excited at 543 nm with a helium–neon laser, and the emission signals were collected at 570–650 nm. TMRM fluorescence was normalized to the minimum fluorescence signal obtained with the mitochondrial uncoupler carbonyl cyanide *p*-(trifluoromethoxy) phenylhydrazone (FCCP, 50 μM).

Ratiometric measurements of mito-ROS were performed using a Leica SP8 confocal with 63 × 1.4 NA oil immersion objective in XYT mode. Cells were imaged in Tyrode solution (see above). MTroGFP-ORP1 was excited at 405 and 488 nm with diode lasers, and the emission signals were collected at 500–530 nm. MTroGFP-ORP1 signals were normalized to the minimum fluorescence signal obtained with the reducing agent dithiothreitol (DTT, 20 mM) and to the fluorescence maximum obtained with the strong oxidizing agent tetramethylazodicarboxamide (diamide, 2 mM) as previously described (Dey et al., 2018).

### Confocal Microscopy Measurements of Intracellular Ca^2+^

Intracellular Ca^2+^ cycling activity in isolated rabbit ventricular myocytes was monitored using a Leica SP5 confocal laser scanning system with a 60 × 1.4 NA oil-immersion objective in XT mode using Ca^2+^-sensitive indicators Fluo-3 (Molecular Probes, Carlsbad, CA, United States), respectively. Cells were loaded with Fluo-3 for 10 min, and after 20 min de-esterification, the dye was excited at 488 nm with an argon laser. Emission was collected at 500–600 nm. CMs were studied in Tyrode solution (see above). Myocytes were paced via field stimulation at 1 Hz using extracellular platinum electrodes. To assess the SR Ca^2+^ load and decay kinetics, 10 mM caffeine was applied at the end of the experiments.

For triggered activity, myocytes were paced at 1 Hz for 30 s, and the latency between the last stimulus in the pacing train and the first SCW was calculated. To assess the effect of mitochondria-derived ROS on CMs from aged rabbits, myocytes were pretreated with the mitochondria-specific ROS scavenger (2-(2,2,6,6-tetramethylpiperidin-1-oxyl-4-ylamino)-2-oxoethyl) triphenylphosphonium chloride (mito-TEMPO, 25 μM, Enzo Life Sciences, Farmingdale, NY, United States) for 10 min. The intracellular Ca^2+^ cycling measurements were performed under β-adrenergic stimulation with 100 nM ISO for HL-1 CMs and 30 nM ISO for aged rabbit CMs.

### Statistical Analysis

Data are presented as mean ± standard deviation (SD) of *n* measured cells or hearts with *n* ≥ 3 biological preparations. Statistical comparisons between groups were performed in OriginPro 2017 with Student’s *t-*test (paired and unpaired) unless otherwise stated. Differences not statistically significant are noted as NS. Differences were considered statistically significant at *p* < 0.05.

## Results

### Autophagy Is Downregulated in the Aging Rabbit Heart

**FIGURE 1:**
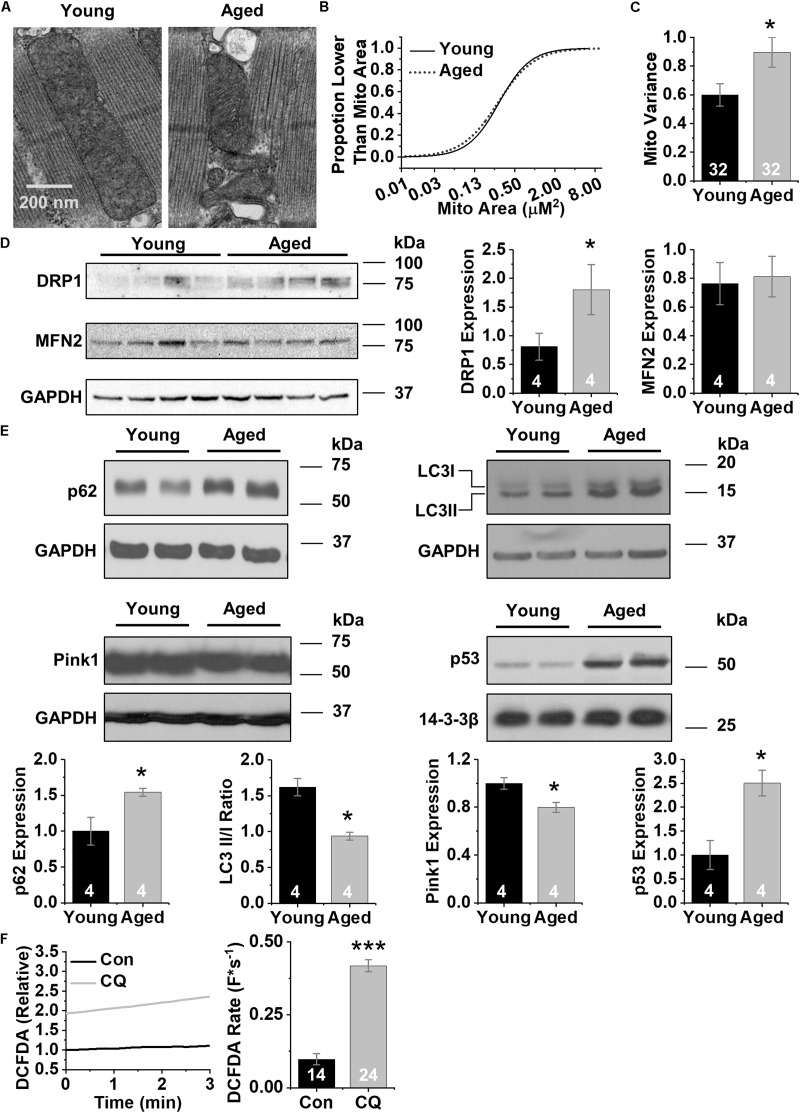
CM aging leads to heterogeneous mitochondria and decreased markers of autophagy. **(A)** Representative transmission electron micrographs of acutely disassociated CMs from young and aged rabbit hearts, scale bar = 200 nm. **(B)** Cumulative distribution of mitochondrial log_2_ cross-sectional areas assessed by image analysis of electron micrographs in young and aged CMs. Analysis of data was performed using the GLIMMIX procedure in SAS Version 9.4 (SAS Inc.) (^∗^*p* < 0.05). **(C)** Variance in mitochondrial cross-sectional areas. **(D)** Representative immunoblots for mitochondrial fission and fusion proteins, DRP1 and MFN2 with densitometry using young and aged whole left ventricular homogenates. **(E)** Representative immunoblots for autophagy (p62, LC3) and mitophagy proteins (Pink1 and p53) using young and aged whole left ventricular homogenates. **(F)** Changes in DCFDA fluorescence in chloroquine- and vehicle-treated CMs. Mean data represent ROS production rate. ^∗^*p* < 0.05, and ^∗∗∗^*p* < 0.001 all values are mean ± SEM.

First we examined the ultrastructure of CMs isolated from both young and aged left ventricular free wall (Cooper et al., 2012, 2013; Morrissey et al., 2017) using transmission electron microscopy. Electron microscopy imaging show that the arrangement of mitochondria in aged CMs is both disorganized and fragmented (Figure 1A). Mitochondrial size analysis was performed using the GLIMMIX procedure in SAS (SAS, Inc.) of 71 sections from four aged and four young hearts (up to 13 sections per heart). Comparing the cumulative distributions of mitochondrial populations revealed a narrower (leptokurtic) curve for young mitochondria and a wider (platykurtic) curve for aged mitochondria (Figure 1B). Further, aged CMs contain a mitochondrial population with increased variance in cross-sectional area compared to young CMs (0.6 vs. 0.9 μm^2^) (*p* < 0.05) (Figure 1C). These observations indicate that mitochondria from aged CMs vary in size to a greater degree than mitochondria from young CMs. We then performed western blot analysis against proteins important in mitochondrial regulation of fission (DRP1) and fusion (MFN2) to better understand why mitochondria in aged CMs are more heterogeneous. Immunoblots with homogenates prepared from left ventricular free walls revealed 2.25-fold higher expression of DRP1 in aged hearts relative to young samples (*p* < 0.05, Figure 1D), but no difference in expression of MFN2. We then performed western blot analysis of homogenates prepared from young and aged hearts against markers of autophagy and mitophagy to understand why defective mitochondria remain in aged CM. The level of the autophagosome scaffolding protein p62 was 1.5-fold higher in aged samples (*p* < 0.05) (Ohsumi, 2001; Hoshino et al., 2013). In addition, the light-chain 3 cleavage ratio (II/I) was 1.7-fold higher in aged compared to young samples (*p* < 0.01), consistent with decreased autophagy. Levels of PINK1, the ubiquitin-mediated mitophagy kinase, were 20% lower in aged relative to young samples (*p* < 0.05). The transcriptional autophagy effector p53 was 2.5-fold more abundant in aged compared to young samples (*p* < 0.05) (Figure 1E). Next, to mimic aging-related reduction in autophagy we administered the autophagy inhibitor chloroquine to young primary rabbit myocytes (600 nM, 3 h). Using the ROS-specific fluorescent indicator DCFDA, we found markedly higher ROS levels as well as ROS production rate (0.19 vs. 0.38 F^∗^s^–1^, *p* < 0.05) (Figure 1F). Overall, we also found that mitochondria in aged CMs have greater variance in mitochondrial cross-sectional area and display protein profiles consistent with decreased autophagy and increased fission. Further, pharmacologically blocking autophagy in CMs from young animals dramatically increased ROS, making the young CMs more closely resemble aged CMs.

### Autophagy Inhibition Decreases Δψm, Increases Mito-ROS, and Promotes Spontaneous Ca^2+^ Release in HL-1 CMs

**FIGURE 2:**
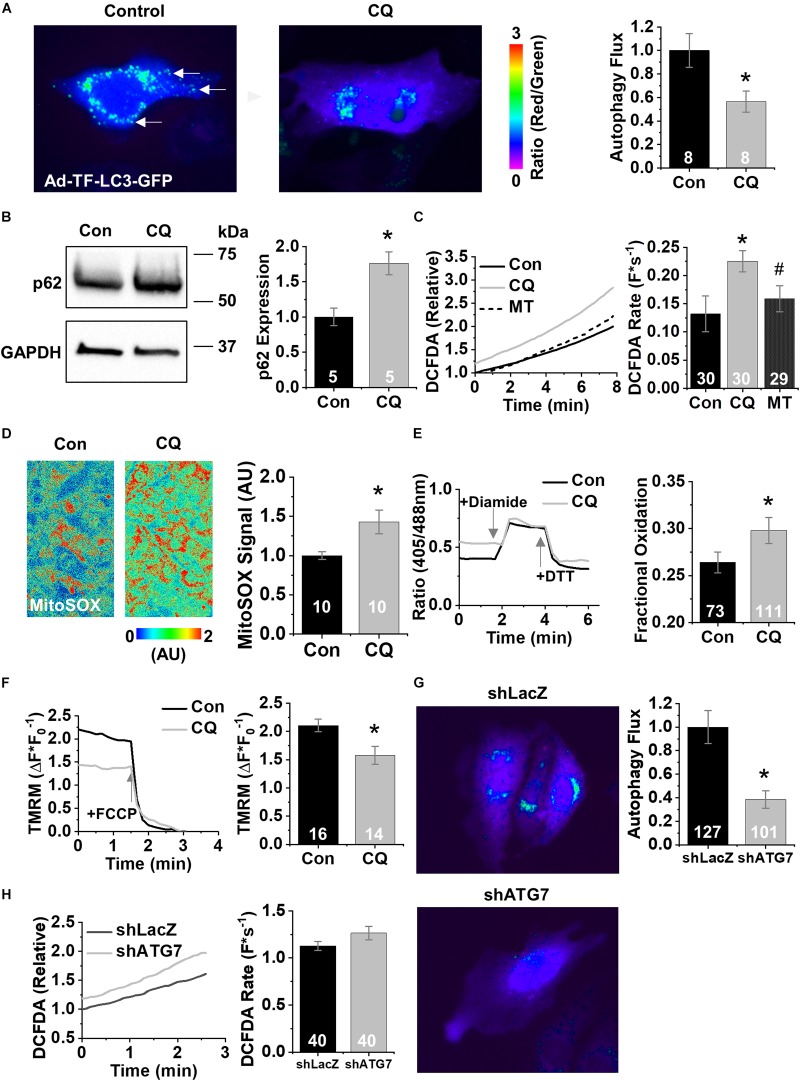
Chloroquine decreases autophagy in HL-1 CMs. **(A)** Fluorescence images of HL-1 CMs infected with Ad-TF-LC3-GFP visualizing autolysosomes as puncta. Examples of autophagosomes are shown by white arrow. **(B)** Representative immunoblots for the autophagy protein p62. **(C)** Changes in DCFDA fluorescence in chloroquine- and vehicle-treated CMs, and from CMs treated with 25 μM mito-TEMPO for 10 min following chloroquine treatment. Mean data represent ROS production rate. **(D)** Representative confocal images of HL1 cells stained with MitoSOX acquired in XYT mode. Relative MitoSOX fluorescence is plotted. **(E)** Representative line traces from confocal imaging of MTroGFP-ORP1. Mean data represent fractional oxidation derived as baseline normalized to fluorescence minimum in the presence of DTT and fluorescence maximum in the presence of diamide. **(F)** Traces and mean data of normalized TMRE fluorescence of chloroquine- and vehicle-treated CMs. Data are normalized to the minimum TMRM fluorescence in the presence of the uncoupling agent FCCP. **(G)** Mean data represent TF-LC3-GFP flux data after treatment with short hairpins against LacZ or ATG7. **(H)** Changes in DCFDA fluorescence after short hairpin treatment. Mean data represent ROS production rate. ^∗^*p* < 0.05, all values are mean ± SEM.

To dissect out the effects of decreased autophagy during cardiac aging on dysfunctional Ca^2+^ handling, we treated cells from the established HL-1 atrial CM cell line (Claycomb et al., 1998) with 600 nM chloroquine or vehicle control for 3 h. At baseline HL-1 CMs produced low levels of mito-ROS. Compared with control cells, chloroquine-treated cells exhibited lower autophagy flux (shown as a high ratio of positive puncta in Figure 2A) using adenovirus containing TF-LC3-GFP. The ratio of red fluorescence (autolysosomes) to green fluorescence (autophagosomes) decreased 44% after the addition of chloroquine, indicating less autophagy. Western blot analysis reveals that these cells expressed 1.75-fold more p62 than did control cells (*p* < 0.05) (Figure 2B). General ROS was measured using the fluorescent indicator CM-H2DCFDA. Chloroquine treatment increased ROS generation compared to controls (0.23 ± 0.06 vs. 0.13 ± 0.09 F^∗^s^–1^) (*p* < 0.05), and ROS generation was rescued by the application of the mito-ROS scavenger mito-TEMPO (0.16 ± 0.07 F^∗^s^–1^) (*p* < 0.05) (Liu et al., 2010; Figure 2C). Direct measurement of mito-ROS using the specific fluorescent superoxide indicator MitoSOX showed that chloroquine treatment induced a 43% increase in mito-ROS (100 ± 20 vs. 143 ± 50%) (Figure 2D, *p* < 0.05). To confirm our results with fluorescent indicator dyes, we used the ratiometric mitochondrial H_2_O_2_ biosensor MTroGFP-ORP1 to specifically measure the level of fractional oxidation due to mitochondrial H_2_O_2_ as previously described (Dey et al., 2018). Mitochondrial H_2_O_2_ levels increased after chloroquine treatment compared to vehicle control (0.26 ± 0.01 vs. 0.30 ± 0.01%, *p* < 0.05) (Figure 2E). Live cell measurements of Δψm using the fluorescence indicator TMRM revealed a chloroquine-mediated decrease in Δψm compared to vehicle control (2.1 ± 0.4 vs. 1.58 ± 0.6 $\Delta \,\text{F}*{\text{F}}_{0}^{-1}$) (*p* < 0.05) (Figure 2F). These data indicate that autophagy blockade in HL-1 CMs decreases Δψm and increases mito-ROS.

**FIGURE 3:**
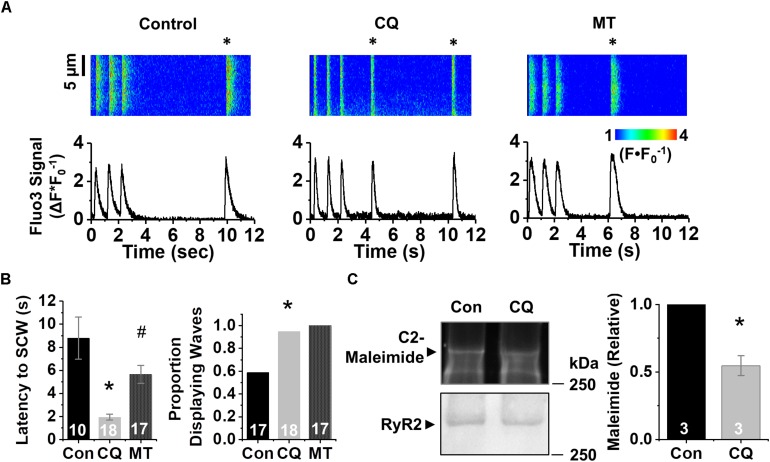
Inhibition of autophagy reduces refractoriness of RyR2-mediated Ca^2+^ release under β-adrenergic stimulation. **(A)** Confocal line scan images and Fluo3 profiles at the end of 1 min 1 Hz pacing train with HL-1 CMs treated with chloroquine or vehicle; all with 100 nM isoproterenol. SCWs are indicated by an asterisk. **(B)** Mean data for SCW latency and proportion of cells exhibiting SCWs (proportion data ^∗^*p* < 0.05, Fisher’s exact test). **(C)** Representative C2-maleimide fluorescence of RyRs from HL-1 CMs treated with chloroquine or vehicle. Relative maleimide fluorescence scored between conditions. *n* = 3 biological preparations from sequential passages. ^∗^*p* < 0.05, all values are mean ± SEM. ^#^*p* < 0.05 compared to control.

To confirm these pharmacological findings, we next tested the effect of genetic ablation of autophagy using short hairpin RNAi (shRNA) against the essential autophagy enzyme ATG7 (shATG7) (Bhuiyan et al., 2013). Treatment of HL-1 CMs with shATG7 resulted in lower autophagy flux compared to that of LacZ control shRNA (1.0 ± 0.14 vs. 0.39 ± 0.07, *p* < 0.05), measured using adenovirus containing TF-LC3-GFP (Figure 2G). HL-1 CMs tolerated the shATG7 treatment, and analysis of ROS production after RNAi showed a strong trend toward ATG7 increasing ROS production (1.13 ± 0.05 vs. 1.26 ± 0.07 F^∗^s^–1^, *p* = 0.058) (Figure 2H). To test the functional effects of decreased autophagy on Ca^2+^ handling, we performed live-cell Ca^2+^ imaging of HL-1 CMs with 1 Hz field-stimulation (Figure 3A). Quantification of Ca^2+^ handling parameters revealed that the proportion of cells exhibiting pro-arrhythmic global SCWs was markedly increased after autophagy block with chloroquine (58% of control cells exhibited SCWs vs. 95% of chloroquine-treated cells, *p* < 0.05) (Figure 3B). Further, the latency at which SCWs formed was significantly shorter with chloroquine treatment compared to control (1.9 ± 1.1 vs. 8.8 ± 5.8 s) (*p* < 0.05). Because multiple physiological changes may be elicited by blocking autophagy with chloroquine, we applied the superoxide scavenger mito-TEMPO (25 μM, 10 min) (Ni et al., 2016) to HL-1 CMs treated with chloroquine. Though there was no change in the proportion of cells exhibiting SCWs, scavenging mito-ROS significantly improved the latency to SCW compared to chloroquine (5.6 ± 3.2 vs. 1.9 ± 1.1 s) (*p* < 0.05).

Given the increase in SCW formation in HL-1 CMs after autophagy block, we assessed the levels of oxidation of the RyR2. Protein lysates conjugated with C2-maleimide (Figure 3C), a free thiol fluorescence indicator (Hanna et al., 2014), were resolved in non-reducing conditions using PAGE. After C2-maleimide fluorescence signal was imaged, immunoblots were performed on the same gel to obtain RyR2 signal, which was in turn used to normalize the C2-maleimide signal against total RyR2. Densitometry showed a twofold reduction in maleimide signal, indicating that blocking autophagy increases thiol oxidation on the RyR2 (*p* < 0.05).

### Increasing Autophagy in Aged Rabbit CMs Restores Δψm, Decreases ROS, and Reduces SCWs

**FIGURE 4:**
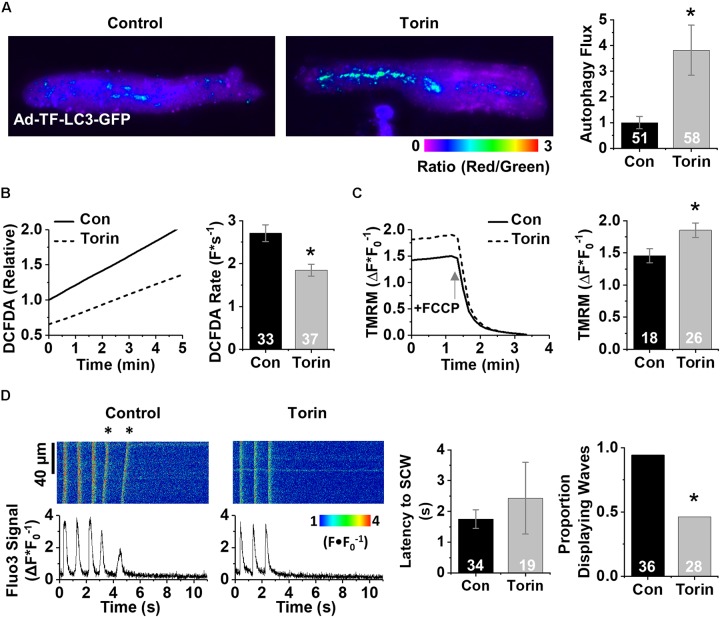
Enhancing autophagy in aged rabbit CMs reduces ROS, restores Δψm, and reverses effects of aging on RyR2-mediated Ca^2+^ release. **(A)** Fluorescence images of aged rabbit CMs treated with Torin1 visualizing autolysosomes as puncta. **(B)** Changes in DCFDA fluorescence in Torin1 and vehicle-treated aged rabbit CMs. Mean data represent ROS production rate. **(C)** Traces and mean data for normalized TMRM fluorescence of Torin1- and vehicle-treated CMs. Data are normalized to the minimum TMRM fluorescence in the presence of the uncoupling agent FCCP (50 μM). **(D)** Confocal line scan images and Fluo3 profiles at the end of 30 s 1 Hz pacing train with aged rabbit CMs treated with Torin1 or vehicle; all with 25 nM isoproterenol. SCWs are indicated by an asterisk. Mean data for SCW latency and proportion of cells exhibiting SCWs (proportion data ^∗^*p* < 0.05, Fisher’s exact test). ^∗^*p* < 0.05, all values are mean ± SEM.

We next treated aged CMs with Torin1 (250 nM, 4 h), an mTOR inhibitor 1000× more selective than rapamycin (Thoreen et al., 2009), to enhance autophagy. TF-LC3-GFP analysis revealed a 3.8-fold increase in autophagy flux after Torin1 treatment (Figure 4A). Aged rabbit CMs treated with Torin1 demonstrated 34% reduction in baseline ROS compared to vehicle control using the general ROS indicator CM-H2DCFDA (100 ± 37.6 vs. 65.9 ± 24.8%) (*p* < 0.01) and a lower ROS production rate (2.71 ± 0.19 vs. 1.85 ± 0.14 F^∗^s^–1^) (*p* < 0.01) (Figure 4B). Enhancing autophagy hyperpolarized Δψm in Torin1-treated CMs compared to vehicle control (1.85 ± 0.57 vs. 1.45 ± 0.46 ΔF^∗^F_0_) (*p* < 0.05) (Figure 4C). To functionally validate the improvement of ROS and Δψm, live cell Ca^2+^ imaging was performed. Representative line scan images after 1 Hz field stimulation are shown in Figure 4D. Strikingly, after enhancing autophagy, there was a 48% reduction in the number of cells displaying pro-arrhythmic SCWs compared to vehicle control (*p* < 0.05, Fisher’s exact test).

**FIGURE 5:**
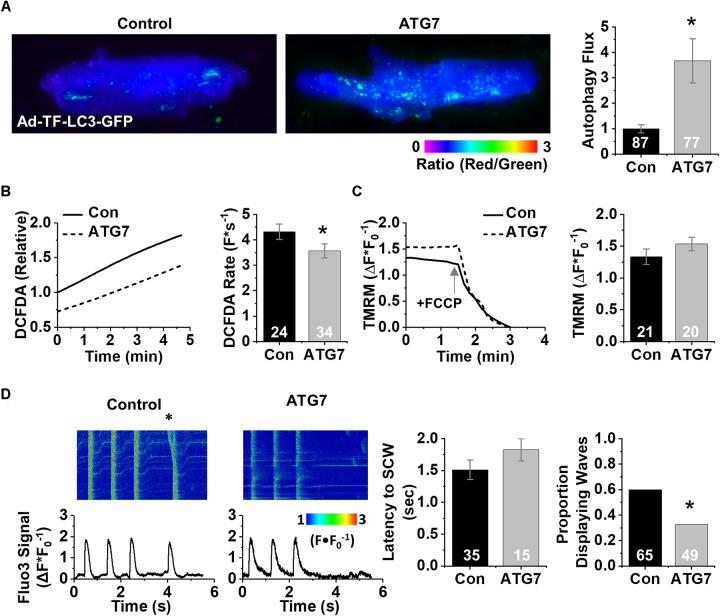
Genetically enhancing autophagy in aged rabbit CMs reduces ROS, restores Δψm, and reverses effects of aging on RyR2-mediated Ca^2+^ release. **(A)** Fluorescence images of aged rabbit CMs infected with Ad-ATG7 visualizing autolysosomes as puncta. **(B)** Changes in DCFDA fluorescence in ATG7 overexpression in aged rabbit CMs. Mean data represent ROS production rate. **(C)** Traces and mean data of normalized TMRM fluorescence of ATG7 and LacZ control-treated CMs. Data are normalized to the minimum TMRM fluorescence in the presence of the uncoupling agent FCCP (50 μM). **(D)** Confocal line scan images and Fluo3 profiles at the end of 30 s 1 Hz pacing train with aged rabbit CMs treated with ATG7 or LacZ control; all with 25 nM isoproterenol. SCWs are indicated by an asterisk. Mean data for SCW latency and proportion of cells exhibiting SCWs (proportion data ^∗^*p* < 0.05, Fisher’s exact test). ^∗^*p* < 0.05, all values are mean ± SEM.

**FIGURE 6:**
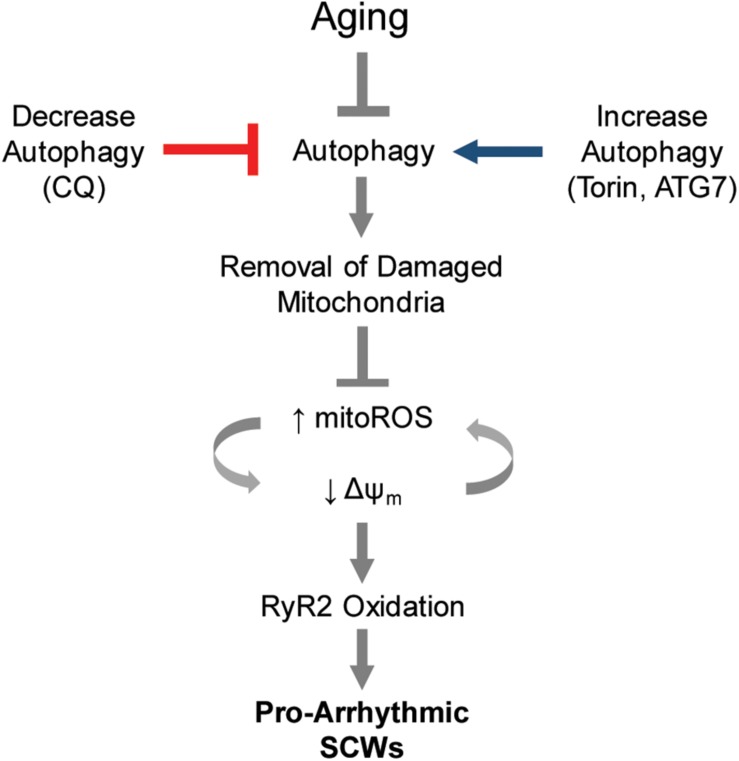
Proposed model of autophagy control of Ca^2+^-mediated cardiac arrhythmia. Autophagy is a major cause of increased mito-ROS production in the aging heart. Blocking or enhancing autophagy is sufficient to modify pathologic mito-ROS production and Δψm during normal aging, thereby controlling the instance of pro-arrhythmic spontaneous Ca^2+^ release via oxidized RyR2s.

Finally, to interrogate the specificity of the pharmacologic experiments, we overexpressed ATG7 in aged rabbit CMs. Autophagy flux was enhanced 3.6-fold in ATG7-overexpressing aged CMs compared to controls (Figure 5A). These cells have less baseline ROS (100 vs. 73%) and a lower ROS production rate (4.31 ± 0.30 vs. 3.56 ± 0.27 ΔF^∗^s^–1^) (*p* < 0.05) (Figure 5B). ATG7 overexpression in treated cells showed a stronger trend toward restored Δψm compared to control cells (1.53 ± 0.11 vs. 1.33 ± 0.12 ΔF^∗^F_0_) (*p* = 0.10) (Figure 5C). Next, we functionally validated the intervention using live-cell Ca^2+^ imaging (representative line scan images after 1 Hz field stimulation are shown in Figure 5D). Similar to Torin1 autophagy enhancement, overexpression of ATG7 resulted in a twofold reduction in the number of cells that displayed pro-arrhythmic SCWs compared to control cells (*p* < 0.05, Fisher’s exact test). *In toto*, these data implicate autophagy as an age-related molecular mechanism that underlies the pro-arrhythmic alterations in mammalian CMs that lead to changes in Ca^2+^ cycling (Figure 6).

## Discussion

Here we show that decreased autophagy during normal aging is one mechanism underlying pro-arrhythmic changes in Ca^2+^ homeostasis in the aging rabbit heart (Figure 6). We demonstrate that: (1) aging hearts contain a more heterogeneous mitochondrial population (assessed by transmission electron microscopy) along with altered levels of autophagy protein compared with young hearts, suggesting decreased autophagy and mitophagy; (2) decreased autophagy in HL-1 cells contributes to Δψm depolarization and excessive generation of mito-ROS capable of oxidizing the SR Ca^2+^ release channel RyR2, leading to increased propensity for pro-arrhythmic SCWs; and (3) enhancing autophagy in aged CMs restores Δψm, decreases mito-ROS, and blocks the formation of SCWs. Our data implicate autophagy as a mechanism underlying age-related increases in mito-ROS production and thereby Ca^2+^-mediated cardiac arrhythmia.

### Autophagy in Aging

Autophagy is generally believed to decrease with age in many organs, especially the heart, as we report here (Hoshino et al., 2013; Lopez-Otin et al., 2013; Siddall et al., 2013; Martinez-Lopez et al., 2015). Since autophagy is a critical regulator of cellular homeostasis, targeting it may be a useful strategy in treating age-related disease. As a cellular regulator, autophagy is responsible for the removal of damaged organelles; with defective autophagy the cell fails to clear damaged mitochondria, which are smaller and produce high levels of ROS. Our data suggest that with aging, mitochondrial size variance increases along with levels of the fission factor DRP1, but there is no change in production of the fusion factor MFN2 (Figure 1). Our assessment of autophagy proteins suggests that autophagy decreases with age, yet LC3-II accumulation may be secondary to either increased autophagosome formation or impaired flux. To that end, we report that the scaffolding protein p62 accumulates during aging which indicates a decrease in lysosomal turnover (i.e., autophagy). Increased p62 expression in autophagy assays is tightly associated with decreased autophagy (Bartlett et al., 2011; Cui et al., 2012). Further, the reduction in LC3II/I ratio suggests a blunted autophagic flux. Decreased Pink1 expression indicates that autophagy/mitophagy machinery is decreased. Lastly, p53 can act as a repressor of autophagy (Tasdemir et al., 2008). The increased expression of p53, in conjunction with the expression profile of the previously mentioned autophagy components suggests that autophagy is decreased in aged hearts (Figure 1; Taneike et al., 2010; Martinez-Lopez et al., 2015).

### Aging Influences Cardiac Mitochondrial Physiology

Several other autophagy components have been reported to decrease with age and can affect mitochondrial physiology and turnover. For instance, the kinase Pink1, which is associated with the E3 ligase Parkin, is decreased during normal cardiac aging and functionally blocks the removal of damaged mitochondria with altered Δψm (Hoshino et al., 2013). Depolarization of the Δψm has been shown to stimulate mito-ROS in smooth muscle cells via SODII and also lead to local spontaneous Ca^2+^-release events known as sparks. Here we use acute pharmacological manipulations to down- or up-regulate autophagy; chloroquine blocks lysosomal acidification and thereby blocks autophagy. Chloroquine is a well-accepted blocker of autophagy. Recent work indicates that chloroquine prevents the fusion of the lysosome to the autophagosome, in addition to the accepted view that it inhibits acidification of the lysosome and thereby protein degradation (Shintani and Klionsky, 2004). Of note, chloroquine may also act via disrupting the Golgi and the endo-lysosomal apparatuses, and these effects could contribute to the interference with lysosome/autophagosome fusion process (Mauthe et al., 2018). The result of both mechanisms is decreased autophagy. In fact, chloroquine is a clinically approved drug which in clinical trials to inhibit autophagy (Towers and Thorburn, 2016). Previous reports showed that short exposure to chloroquine (2–6 h) led to decreased autophagy flux (Iwai-Kanai et al., 2008); therefore, we treated our HL-1 CMs with 600 nM chloroquine for 3 h. Chloroquine decreased autophagy flux as measured by the indicator TF-LC3-GFP and led to accumulation of p62 polypeptides (Figure 2). Functionally, our manipulation increased mito-ROS and depolarized the Δψm (Figure 2). Genetic manipulation of autophagy using shRNA against the critical autophagy protein ATG7 led to a drastic reduction in autophagy measured by TF-LC3-GFP. Despite the large depletion of ATG7, a trend of increased total ROS was observed. Although this increase in ROS is not statistically different from control conditions, the pattern is consistent with our observation that chloroquine decreases autophagy and increases ROS (Figure 2).

### Age-Related Dysfunctional Ca^2+^ Release Underlies Increased Arrhythmic Potential

Cardiac contraction relies on Ca^2+^ release from intracellular Ca^2+^ stores mediated by the SR Ca^2+^ release channel RyR2. In cardiac disease or aging, RyR2 becomes more active, which likely helps maintain cardiac output to meet baseline metabolic demands (Cooper et al., 2013). However, during stress, hyperactivity of RyR2s has been associated with SCWs, which could translate into disturbances in membrane potential, i.e., delayed or early after depolarizations leading to malignant arrhythmias and sudden cardiac death (Belevych et al., 2012). Posttranslational modifications of the channel, including phosphorylation by PKA and/or CaMKII and oxidation of reactive cysteines, have been implicated in the RyR2 hyperactivity characteristic of cardiac disease and aging (Belevych et al., 2012; Zima et al., 2014; Hamilton and Terentyev, 2018, 2019). Consistent with this, treatment with ROS scavengers stabilized aberrant Ca^2+^ release and lessened triggers for arrhythmia in conditions accompanied by oxidative stress, including heart failure (Terentyev et al., 2008) and myocardial infarction (Belevych et al., 2012). Likewise, we previously reported that in aging rabbit ventricular CMs, the scavenging of mito-ROS restores the oxidative state of RyR2s, stabilizing Ca^2+^ release and resulting in fewer SCWs in response to β-adrenergic stimulation to mimic stress (Cooper et al., 2013). Ca^2+^ imaging revealed alterations in several key parameters such as increased propensity for spontaneous Ca^2+^ release (measured by decreased latency to spontaneous wave after pacing train), and a greater proportion of cells after autophagy block that displayed spontaneous activity (Figure 3). Importantly, we found that this dysfunction is rescued by scavenging mito-ROS via application of mito-TEMPO. These data are in line with our previous report that mito-ROS oxidizes the RyR2 during aging, leading to increased SR Ca^2+^ leak and generation of SCWs (Cooper et al., 2013). Targeted enhancement of autophagy effectively stabilized RyR2-mediated Ca^2+^ release via downregulation of mito-ROS production (Figures 4, 5).

### Targeting Mito-ROS in Aging to Normalize Aberrant Ca^2+^ Handling

Mitochondria participate in numerous processes including bioenergetics, intracellular signaling, and immune response (Chen et al., 2007; Nakai et al., 2007; Sovari et al., 2013; Cordeiro et al., 2015). Previous reports implicate mTOR not only as an important signaling intermediate between the mitochondria and nucleus that helps maintain mitochondrial function, but also a critical regulator of autophagy (Matsui et al., 2007; Taneike et al., 2016). Our data show that inhibition of mTOR by Torin1 improves aged CM physiology by decreasing ROS production and hyperpolarization of depolarized Δψm in the aging CM (Figure 4), in accordance with other reports (Matsui et al., 2007; Bitto et al., 2010; Lerner et al., 2013). Ca^2+^ imaging of Torin1-treated aged CMs reveals a twofold decrease in the number of cells exhibiting pro-arrhythmic SCW activity (Figure 4). Similar results were obtained in experiments with overexpression of the autophagy effector ATG7 (Figure 5). A three- to fourfold overexpression of ATG7 significantly increased autophagy flux and decreased ROS. Although not statistically significant, ATG7 overexpression hyperpolarized the mitochondrial membrane potential 15%. Indeed, ATG7-overexpressing aged CMs demonstrate twofold fewer cells exhibiting pro-arrhythmic SCW activity. The physiological changes observed after ATG7 overexpression are consistent with those are Torin1 stimulation of autophagy (Figure 4), namely increased autophagy flux, decreased ROS, increased mitochondria membrane potential, and decreased instance of SCW activity. Taken together, our results implicate diminished autophagy as a key contributor to increased mito-ROS and correspondingly pro-arrhythmic RyR2-mediated spontaneous SR Ca^2+^ release in aged CMs. Restoration of autophagy may be a rational strategy to reduce arrhythmic risk associated with aging.

## Conclusion

We demonstrate that enhancing autophagy via acute application of Torin1 or ATG7 overexpression in primary CMs from aged rabbits effectively mimics the effects of ROS scavengers at the cellular level, reducing mito-ROS and effectively stabilizing RyR2-mediated Ca^2+^ release (Figures 4, 5). Conversely, blocking autophagy in HL-1 CMs promotes the oxidation of RyR2 and ROS-dependent pro-arrhythmic disturbances in Ca^2+^ handling, as manifested in an increased propensity for generation of pro-arrhythmic SCWs (Figure 3). In combination with our previously published results, our current data suggest autophagy (Figure 6) as a target for modulation of mito-ROS in the aging mammalian heart to prevent Ca^2+^-dependent arrhythmia.

## Data Availability Statement

All datasets generated for this study are included in the manuscript/supplementary files.

## Ethics Statement

This work conformed to the current Guide for Care and Use of Laboratory Animals published by the National Institutes of Health (2011) as well as the American Physiological Society (“Guiding Principles for Research Involving Animals and Human Beings”) and was approved by the Institutional Animal Care and Use Committee at Rhode Island Hospital. All experiments were carried out in the Cardiovascular Research Center of the Cardiovascular Institute at Lifespan or Johns Hopkins Medical Institute.

## Author Contributions

KM, DT, JS, and GK contributed to the conception and design of the study. KM, JO-U, and DT worked on interpretation of data and writing of the manuscript. KM, LC, BB, YL, and DT performed the experiments and analyzed the results. All authors read and approved the final submission of the manuscript.

## Conflict of Interest

The authors declare that the research was conducted in the absence of any commercial or financial relationships that could be construed as a potential conflict of interest.
